# Copper-Arsenic-Sulfide Thin-Films from Local Raw Materials Deposited via RF Co-Sputtering for Photovoltaics

**DOI:** 10.3390/nano12193268

**Published:** 2022-09-20

**Authors:** Pedro Centeno, Miguel Alexandre, Filipe Neves, Elvira Fortunato, Rodrigo Martins, Hugo Águas, Manuel J. Mendes

**Affiliations:** 1 CENIMAT|i3N, Department of Materials Science, School of Science and Technology, NOVA University Lisbon and CEMOP/UNINOVA, 2829-516 Caparica, Portugal; 2 LNEG, Laboratório Nacional de Energia e Geologia, Estrada do Paço do Lumiar 22, 1649-038 Lisboa, Portugal

**Keywords:** photovoltaic materials, thin-film semiconductors, copper arsenic sulfide compounds, RF co-sputtering

## Abstract

The inexorable increase of energy demand and the efficiency bottleneck of monocrystalline silicon solar cell technology is promoting the research and development of alternative photovoltaic materials. Copper-arsenic-sulfide (CAS) compounds are still rather unexplored in the literature, yet they have been regarded as promising candidates for use as p-type absorber in solar cells, owing to their broad raw material availability, suitable bandgap and high absorption coefficient. Here, a comprehensive study is presented on the structural and optoelectronic properties of CAS thin-films deposited via radio-frequency magnetron co-sputtering, using a commercial Cu target together with a Cu-As-S target with material obtained from local resources, specifically from mines in the Portuguese region of the Iberian Pyrite Belt. Raman and X-ray diffraction analysis confirm that the use of two targets results in films with pronounced stoichiometry gradients, suggesting a transition from amorphous CAS compounds to crystalline djurleite (Cu_31_S_16_), with the increasing proximity to the Cu target. Resistivity values from 4.7 mΩ·cm to 17.4 Ω·cm are obtained, being the lowest resistive films, those with pronounced sub-bandgap free-carrier absorption. The bandgap values range from 2.20 to 2.65 eV, indicating promising application as wide-bandgap semiconductors in third-generation (e.g., multi-junction) photovoltaic devices.

## 1. Introduction

Clean, decarbonized energy is crucial for the sustainability of the modern world. It is, therefore, needed a continuous effort in the development and employment of innovative, renewable technologies and materials heading towards zero carbon emissions. Photovoltaic (PV) technology represents a strong contender in reaching these goals, as it virtually has no carbon footprint stemming from energy generation.

Several thin-film (TF) PV technologies, such as perovskite, Cu(In,Ga)Se_2_ (CIGS) and CdTe solar cells are already in sight of competing with the monocrystalline silicon (c-Si) cells’ record of 26.7% efficiency [1], registering 25.5% [2], 23.35% [3] and 22.1% [4] efficiency, respectively. TF technologies benefit from having 1% or even 0.1% the thickness of c-Si wafer-based PV cells, which allows for lightweight and flexibility, opening up a wide range of applications such as in solar-powered mobility, IoT and even wearables. In addition, as a means to circumvent the reduced broadband absorption of the thin PV materials, several advanced photonic strategies are being developed to enhance the optical density of TF cells via light-trapping [5,6,7,8,9,10,11], thus pointing the way towards efficiencies at the level of the thick-wafer PV devices.

However, some TF PV technologies suffer from critical limitations that encumbers their mass production and commercialization, such as the reduced stability of perovskite cells [12], the limited supply of In for CIGS [13] and the toxicity of Cd in CdTe and Pb in perovskite [14]. Extensive research efforts have been employed to develop Cu_2_(Zn,Sn)S_4_ (CZTS) and Cu_2_(Zn,Sn)Se_4_ (CZTSe) absorbers as an attempt to address the aforementioned concerns [15]. Nonetheless, the complex quaternary nature of these chalcogenide compounds poses problems regarding high density trap states and band tailing [16,17], having thus far enabled only 12.6% efficiency [18].

Ternary CAS compounds are relatively earth-abundant, p-type semiconductor materials that can be found in their mineral form in nature [19], often being regarded as mining by-products. These compounds, still in their research infancy, have emerged as a promising candidate for use in thermoelectrics [20], PV [13,21,22] and other applications [23]. There are six main phases in which CAS compounds can be found: lautite (CuAsS), sinnerite (Cu_6_As_4_S_9_), tennantite (Cu_12_As_4_S_13_), tetragonal luzonite (Cu_3_AsS_4_), orthorhombic enargite (Cu_3_AsS_4_) and “compound A” (Cu_24_As_12_S_31_). Apart from lautite and “compound A”, for which information is scarce, CAS compounds were reported to have semiconductor properties comprehending bandgaps between 1.19 and 1.75 eV with high absorption coefficients (~10^5^ cm^−1^). Namely, a bandgap of 1.2 eV has been determined for luzonite [24] and for sinnerite [21,24], 1.24–1.43 eV for enargite [21,25,26] and 1.75 eV for tennantite [27]. Presently, these CAS compounds have mainly been synthesized as nanocrystals through solution-based methods [27,28,29].

So far, the highest attained efficiency was 0.49% by Andler et al. [30], with an enargite-based PV cell obtained through a heat treatment of copper-sulfide films under an As- and S-rich atmosphere. The low efficiency has been attributed to film porosity, presence of unwanted secondary phases and poor band alignment with the CdS (n-type semiconductor) selective contact [13,30,31].

Additionally, several extraordinary circumstances can impact the cost and, in more extreme cases, the availability of raw materials. For instance, the now endemic COVID-19 and the current geo-political situation in Eastern Europe are events that have raised awareness and urgency concerning the need for local, renewable resources. One way to address this issue is the recovery of raw materials from mining waste, turning an environmental problem into an opportunity to recover valuable resources, while contributing to a more circular economy.

Following a previous study [32], we report on low-cost co-sputtered CAS TFs, using a commercial Cu target and a Cu-As-S target composed of by-product material from the Cu exploration at the Barrigão mine located in the Portuguese region of the Iberian Pyrite Belt. Radio-frequency (RF) sputtering is a very well established physical vapor deposition technique used in a wide range of optoelectronic applications [33,34]. This technique enables the use of various materials, ensuring high deposition rates, homogeneity in large areas and reproducibility [35]. We show the potential of depositing these compounds via RF magnetron co-sputtering for TF PV technologies, which enables the tunability of their optoelectronic properties through careful management of their stoichiometry.

## 2. Materials and Methods

**Figure 1:**
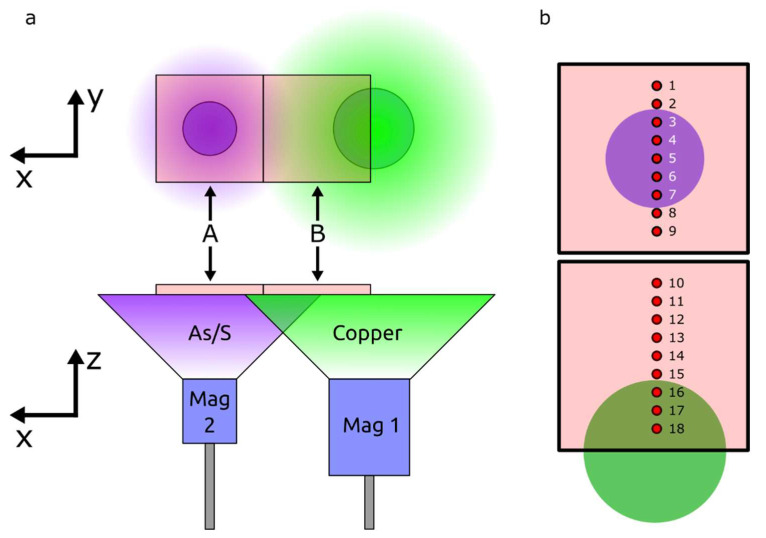
(**a**) Illustration of the co-sputtering system configuration, displaying the relative position of each target and the two glass substrates, from two perspectives (top and side view). The sketch in (**b**) shows the position of the 18 measurement points along two glass substrates, with the indication of the relative position of the targets.

The TF deposition was carried out on 10 cm × 10 cm soda-lime glass substrates, as depicted in Figure 1. The glasses had previously been cleaned resorting to a sequential sonication in acetone and isopropanol for 15 min each, followed by rinsing in ultrapure water. Finally, they were dried under a nitrogen flow. RF magnetron co-sputtering was used to deposit the CAS TFs using a commercial 3-inch Cu target and a home-made 2-inch Cu-As-S target, both normally facing the glass substrate.

The raw material (ore) for the Cu-As-S target was collected from the Barrigão mine dumps, located in the Portuguese region of the Iberian Pyrite Belt. The ore was crushed and ground to a fine powder in a vibratory disc mill. This powder was then pressed with 20–25 tf at room temperature, resulting in a 5 mm-thick, 2-inch diameter sputtering target. Finally, this target was subjected to sinterization at 400 °C for a period of 12 h under a nitrogen-rich atmosphere. The composition of the target was verified by energy dispersive spectroscopy (EDS).

As shown in Figure 1b, the Cu-As-S target was centered with one glass substrate and the Cu target was centered in the far edge of the adjacent substrate. The composite film analyzed in this work was deposited with previously-optimized conditions consisting in an Ar flow rate of 20 sccm at a pressure of 1.0 mTorr. The power applied both to the Cu and the Cu-As-S targets was 50 W.

The characterization measurements were performed on a set of specific points along the film, spaced 1 cm from each other, as shown in Figure 1b.

To obtain the values of films’ thickness, an Ambios Technology XP-200 profilometer (Santa Cruz, CA, USA) was used. The measurements were digitally acquired with XP-Plus Stylus Profilometer software (Version 1.0.20.254, Ambios technology, Milpitas, CA, USA).

Using a Hitachi TM3030 Plus system (Tokyo, Japan), EDS and scanning electron microscopy (SEM) analyses were performed to determine the atomic concentration and to verify the morphology along the films, respectively.

Raman spectroscopy measurements were carried out using a Renishaw inVia Qontor micro-Raman spectrometer (New Mills, UK) equipped with an air-cooled charge-coupled device (CCD) detector and an He–Ne laser operating at 50 mW of 532 nm laser excitation. The spectral resolution of the spectroscopic system is 0.3 cm^−1^. The laser beam was focused with a 100× Leica objective lens (N Plan EPI) (Wetzlar, Germany) with a numerical aperture of 0.85. All of the measurements were recorded in the 60–1800 cm^−1^ range with an integration time of 10 scans of 10 s each in order to reduce the random background noise induced by the detector, without significantly increasing the acquisition time. The intensity of the incident laser was 2.5 mW. Between different Raman sessions, the spectrograph was calibrated using the Raman line at 521 cm^−1^ of an internal Si wafer for reducing possible fluctuations of the Raman system. All the raw data were collected digitally with Wire 5.0 software for processing.

X-ray diffraction measurements were performed in a parallel beam grazing-incidence configuration (GIXRD) with a PANalytical’s X’Pert PRO MPD diffractometer (Almelo, The Netherlands) with CuKα radiation and using an X’Celerator 1D detector (rotated at 90° to a 0D configuration). The GIXRD data were recorded at optimized incidence omega angles of 0.4°, 1° and 1.5°, with a 2θ scan range from 10 to 70 and a step size of 0.05°.

Transmission and reflection measurements were performed using a Perkin Elmer Lambda 950 UV-Vis-NIR spectrophotometer (Waltham, MA, USA), equipped with a 15 cm diameter integrating sphere, in the wavelength range of 300–1500 nm.

The resistivity values were calculated by multiplying the previously measured thickness by the sheet resistance (R_S_) values, that were acquired using a JANDEL 4-point probe system, through the application of 1 µA current on the sample and measuring the resulting voltage drop, according to Ohm’s law. The conductivity of the films was measured for substrate temperatures from 300 K to 390 K. The activation energy (E_A_) was calculated resorting to Equation (1):

(1) $$\sigma =\sigma_{0}e^{-\frac{E_{A}}{k_{B}T}}$$

where σ is the conductivity, *T* is the substrate temperature, $\sigma_{0}$ is a parameter representing the conductivity for T→∞ and $k_{B}$ is the Boltzmann constant.

## 3. Results and Discussion

As shown in Figure 1a, the co-sputtering deposition system used in this work held two parallel magnetrons normally facing the substrates, one equipped with a 3-inch commercial Cu target and another one equipped with a home-made 2-inch Cu-As-S target. However, the Cu-As-S target had not only Cu, As and S in its composition, but also SiO_2_ and traces of Sn and Fe, as shown in Figure S1 in Supplementary Materials (SM).

Preliminary tests showed that depositions with solely the Cu-As-S target, mainly resulted in As-S films, thus, from this point onwards, we refer to the 2nd target simply as As-S. To address this Cu deficiency, a Cu target was added to the system, which ultimately became a very important feature in this study, allowing a careful tuning of the optoelectronic properties of the deposited material. Therefore, the resulting CAS TFs analyzed in this study predominantly present a pronounced gradient throughout the deposition length. The structural, optical and electronic properties of the as-deposited CAS TFs were assessed on 18 equally-spaced points across each substrate, as shown in Figure 1b.

### 3.1. Morphological and Structural Assessment

**Figure 2:**
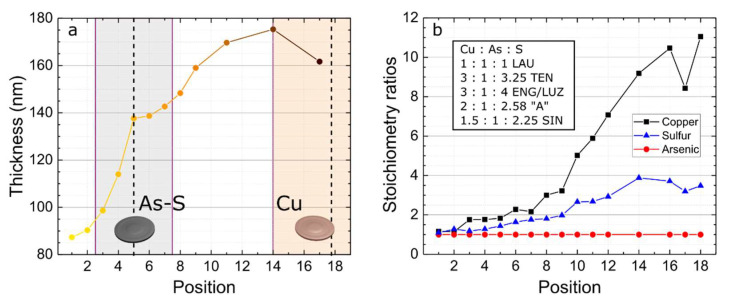
(**a**) Film thickness obtained by profilometry. The colored areas of the plot illustrate the positions of the substrate that are directly above each target (As-S in grey, Cu in orange) and the dashed vertical lines denote the center of the target (**b**) Stoichiometry ratios of Cu/As, S/As and As/As obtained by EDS with inset table presenting the various known phases of CAS.

Figure 2a shows the thickness registered by profilometry at each point of the CAS TFs. A steady increase is observed going from point 1 to 14 from the higher Cu deposition rate, in contrast to the lower As-S deposition rate. Furthermore, the Cu deposition rate seems to be quite uniform in the area directly above the respective target, as from point 14 to 18, the slight decrease in thickness can be attributed to the decreasing contribution from the As-S deposition.

To provide a direct comparison with the known CAS phases, the EDS results presented in Figure 2b show the Cu-As-S stoichiometry in each position of the as-deposited CAS TF, in which the atomic concentrations of Cu, As and S are normalized to that of As, being the absolute values presented in Figure S2 in SM. The results generally point to an exponential-like increase of Cu concentration in relation to As from position 1 to 18, due to the increasing proximity to the Cu target. This correlation is in agreement with a preliminary study concerning a Cu-only TF deposited in the same conditions as in the CAS TF, as shown in Figure S3a in SM. Initially, the Cu/As ratio slowly increases from position 1 (ratio of 1.16) to position 7 (ratio of 2.16), resulting in an average increase of ~0.19 cm^−1^. Then, the Cu/As ratio peaks on position 18, with a value of 11.05, demonstrating a more abrupt increase of ~0.78 cm^−1^. This difference is attributed to the Cu deposition distribution, which is relatively low in regions of the substrate far from the Cu target and becomes much more intense when that distance is shrunken. This distribution could be further tuned through the adjustment of the substrate height [36]. It should be noted that the relatively lower Cu/As ratio in position 17 can be attributed to a slight EDS measurement inaccuracy, as it does not reflect any significant change observed in the physical properties of the films in the following results.

Figure S3b in SM shows the S/As atomic concentration ratio of a preliminary study on a 10 cm × 10 cm arsenic sulfide TF. It shows a lower relative concentration of S than that of As, as well as a symmetric distribution around the sample’s center (point 5). While it would be expected to have a similar S/As ratio profile in the CAS TF, the results reveal a different scenario. Contrary to the arsenic sulfide TF, the S concentration is higher than that of As in the CAS TF, which could be related to a preferential incorporation of S over As, due to the electrochemical affinity with Cu (As^3+^, Cu^2+^, S^2−^). With the exception of positions 1 (with Cu:As:S stoichiometry of 1.16:1:1.09) and 2 (1.15:1:1.27), which register stoichiometries that are very similar to that of lautite (1:1:1), none of the measured points of the deposited CAS TF seem to directly match a known CAS phase, because the majority of the film has a Cu-rich content, especially from position 10 to 18 (closer to the Cu target).

**Figure 3:**
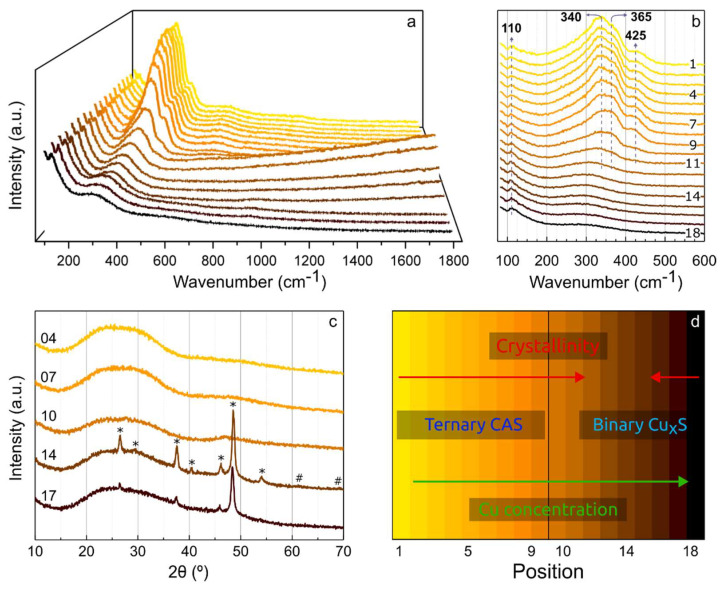
(**a**,**b**) Raman and (**c**) GIXRD (Omega = 1°) spectra of several points measured along an as-deposited 20 × 10 cm thin-film with variable atomic concentrations of Cu, As and S. Position 1 and 18 are the furthest from and closest to the Cu target, respectively, as depicted in Figure 1. (**d**) Representation of the main material attributes of the deposited thin-films.

Figure 3 shows the Raman and the GIXRD spectral patterns of the points measured across the as-deposited CAS film and a representation of the main attributes of the deposited TFs. Figure 3a shows that Raman bands are present between 100 and 800 cm^−1^ throughout the sample. Despite no additional bands being detected above 800 cm^−1^, it is worth noting that from position 10 to 15, there is an indication of fluorescence occurrence [37], with highest relative intensity in position 11. A sketch of the variation of the structural composition of the deposited film, inferred from the presented results, is shown in Figure 3d to assist in the conceptual understanding.

In position 1 (furthest from Cu target), an asymmetric broad band can be observed at ~340 cm^−1^, with a conspicuous shoulder at 425 cm^−1^. A weak band at ~110 cm^−1^ and a very broad weak band from 530 to 800 cm^−1^ are also present. At first glance, there appears to be no appreciable change from position 1 to 6, but, upon closer inspection at the 340 cm^−1^ band, in Figure 3b, a subtle narrow band seems to appear at 365 cm^−1^ with increasing intensity, relatively to the one in 340 cm^−1^. Moreover, the low degree of crystallinity, given by the relatively broad FWHM of the bands, allows bond angles and lengths to occur randomly, enabling slight shifts in the Raman band positions [38]. Together with the spectra presented in Figure S4 in SM, that show reference Raman spectral patterns for CAS compounds, these results suggest that multiple CAS phases (enargite—RRUFF R070176, luzonite—RRUFF R060390 and tennantite—RRUFF R110024), with special emphasis on enargite, are present and evolving as the concentrations of Cu and S increase, with respect to As.

From position 6 to 12, the intensity of all bands, except that at 110 cm^−1^, seems to progressively decrease, leaving solely a broad band from 200 to 400 cm^−1^, which aligns well with djurleite (Cu_31_S_16_—R070333). This band extends to the end of the sample (position 18). The band at 110 cm^−1^ remains unaltered throughout the 18 positions.

The GIXRD spectra of five positions (4, 7, 10, 14 and 17), measured at an incidence angle omega = 1.0°, are shown in Figure 3c. The results reveal that the TF is highly amorphous from position 1 (furthest from the Cu target) to 10. However, contrary to position 18, of Figure S4 in SM, which detects only the contribution of the glass substrate, a broad “bump” is visible at around 2θ = 30°, which progressively fades from position 1 to 14. Additionally, a small peak at 47° seems to appear from position 4 to 10, suggesting the formation of different compounds and an increasing degree of crystallinity. The comparison with the reference XRD spectra of enargite, luzonite and tennantite of Figure S4a in SM, that displays peaks around 2θ = 30° and 50°, supports the assumption stated in the Raman analysis, in which one or more CAS phases are present between positions 1 and 6.

When the Cu concentration becomes sufficiently high, the conditions for the occurrence of a ternary CAS compound cease, and in the GIXRD spectrum of position 14 in Figure 3c, it is clearly shown the formation of a crystalline binary copper-sulfide phase, entitled djurleite (Cu_31_S_16_) [39]. The diffraction peaks of Cu_31_S_16_ (marked with “*”) can be observed at 2θ = 26.5°, 29.3°, 37.6°, 40.4°, 46.1°, 48.7° and 54.0°. Although it is not clear with Omega = 1.0°, GIXRD measurements with Omega = 0.4° and 1.5°, shown in Figure S4c,d in SM, evidence peaks at 61.3° and 69.1° (marked with “#”), which also correspond to Cu_31_S_16_. The abnormally high intensity of the peak at 48.7° is due to the preferred crystal growth orientation corresponding to the (−282) plane [40], which is a known characteristic of the sputtering deposition process [41].

The GIXRD spectrum of position 17 details a small shift of several peaks to lower 2θ values, which is characteristic of the increase of Cu concentration for these copper sulfide compounds [39,42], and a general decrease of peak intensity, thus indicating reduced crystallinity. Nevertheless, the main phase is still considered to be Cu_31_S_16_ [42,43]. Moreover, the GIXRD spectrum of position 18 (furthest from the As-S target), shown in Figure S4d in SM, presents no peaks, revealing the complete amorphization of the film. However, since both the EDS and Raman results remain practically unaltered, it is expected that a marginally Cu richer phase of copper sulfide is attained.

### 3.2. Optical and Electrical Assessment

**Figure 4:**
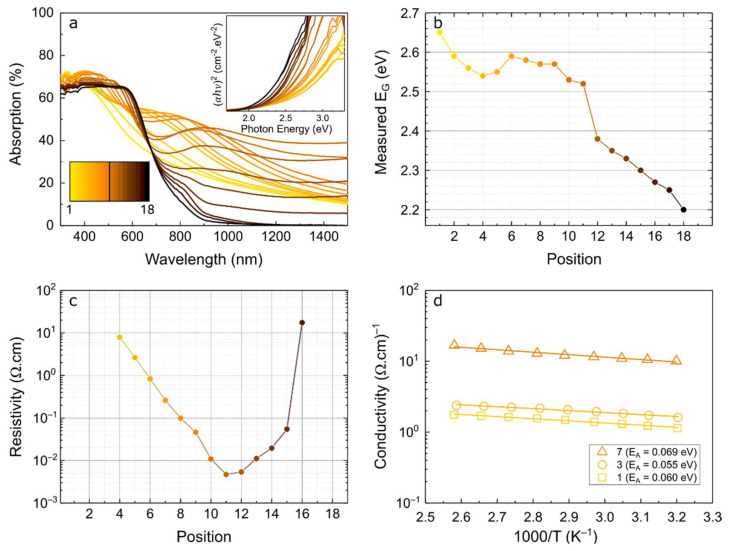
(**a**) Absorption spectra of the 18 positions calculated from the measured transmission and reflection results, by A = 100%–T–R. Inset with Tauc plot of each position, calculated for direct bandgap semiconductors; (**b**) Bandgap calculated for each position in the substrate; (**c**) Resistivity values across the 18 positions; (**d**) Temperature dependence of the conductivity of positions 1, 3 and 7, and respective activation energies given by the slope of the linear fits, represented by solid lines. Position 1 and 18 are the furthest from and closest to the Cu target, respectively.

Figure 4 shows the optical absorption with the calculated bandgap values via a Tauc plot analysis [44], as well as the experimental resistivity and temperature-dependent conductivity. The absorption spectra were determined from the total transmission (T_T_) and reflection (R_T_) measurements, shown in Figure S5 in SM, at the different positions along the sample. Figure 4a shows that the absorption of the film is higher than 60% in the visible range and that the absorption onset wavelength increases from ~400 nm (position 1) to ~600 nm (position 18) along the TF. The absorption spectra of positions 10 to 15 show extended absorption into the near infrared region, due to free-carrier absorption arising from the formation of binary copper sulfide compounds. Moreover, both the intensity and wavelength range of the free-carrier absorption increase with decreasing Cu/As ratio, which is in agreement with previous reports on copper sulfides [40,45]. The bandgap values shown in Figure 4b, calculated from the inset Tauc plots of Figure 4a, reveal that the increase of Cu concentration, relatively to As and S, generally results in bandgap decrease. Nonetheless, the optical bandgaps obtained for the positions with amorphous CAS compounds (1 through 9) are relatively high, compared with other values reported in the literature [21,24,25,26,27].

In what concerns the electrical measurements, the results of Figure 4c display a variation of resistivity from ~10^1^ Ω·cm (both in the Cu-poor and Cu-rich extremities of the TF) to ~4.5 × 10^−3^ Ω·cm (in the region where copper sulfide withlow Cu/S ratio is formed), which agrees well with the results previously provided in this study. In addition, to verify the semiconductor behavior of the ternary CAS compounds, temperature-dependent conductivity measurements of points 1, 3 and 7, shown in Figure 4d, were performed. It can be observed that the increase of Cu in the films’ compositions results in higher conductivity, as expected, registering 1.07, 1.52 and 8.76 Ω^−1^·cm^−1^ at 300 K for positions 1, 3 and 7, respectively. However, the activation energy (E_A_), given by the slope of the linear regression to Equation (1), is rather low and similar at the three positions, which is indicative of a highly doped semiconductor, probably due to incorporation of oxygen impurities that were present in the target material, as shown in Figure S1 in SM.

Although the amorphous CAS semiconductors here reported show high bandgap, this study unravels the feasibility of the fabrication of alternative semiconductor materials through RF magnetron co-sputtering for application in high-efficiency solar cells. Furthermore, the facile tunability of the deposition processing parameters allows for the optimization of these CAS layers for single junction (low bandgap) or multijunction/tandem PV devices (intermediate-to-high bandgap).

## 4. Conclusions

In this work, we have explored novel wide-bandgap semiconductor materials with potential interest for PV applications, based in amorphous copper-arsenic-sulfide thin-films successfully deposited via radio-frequency magnetron co-sputtering. For that, a commercial Cu target was paired with a Cu-As-S target, composed of by-product material from the Cu exploration at the Barrigão mine located in the Portuguese region of the Iberian Pyrite Belt. Due to the system configuration, binary copper sulfide phases with high degree of crystallinity were also formed in a substantial area of the substrates. Although the obtained bandgap values of ~2.5 eV for the CAS compounds ultimately result in the absorption of a fairly limited wavelength range, the tunability of the co-sputtering deposition parameters potentiates the optimization of the stoichiometry of these films, as demonstrated in this study, thus enabling the tuning of the bandgap according to the intended application.

## Data Availability

Not applicable.

## Supplementary Materials

The following supporting information can be downloaded at: https://www.mdpi.com/article/10.3390/nano12193268/s1, Figure S1. (a) SEM image of the copper-arsenic-sulfide target used in the deposition of the CAS films and (b–h) respective EDS color map images of the target displaying its content: Copper, arsenic and sulfur with SiO_2_, Fe and Sn impurities. The scale bar is common to all images; Figure S2. Atomic percentages of Cu, As and S in each position within the thin-film; Figure S3. (a) Cu atomic percentage obtained in a preliminary 20 by 10 cm Cu thin-film, deposited with the same parameters of the main deposition (50 W, 50 sccm Ar flow, 1.0 mTorr). (b) S/As Ratio of the preliminary 10 by 10 cm As-S thin-film, deposited with the same parameters of the main deposition (50 W, 50 sccm Ar flow, 1.0 mTorr). The positions presented correspond to those illustrated in Figure 1 of the main article; Figure S4. (a) XRD reference spectra of two copper sulfide phases (roxbyite and djurleite) and three copper arsenic sulfide phases (enargite, luzonite and tennantite). (b) Raman reference spectra of djurleite and three copper arsenic sulfide phases (enargite, luzonite and tennantite). GIXRD spectra of five positions within the deposited thin-film with Omega = 0.4° (c) and 1.5° (d); Figure S5. Measured transmission (a) and reflection (b) spectra of positions 1 (light yellow) to 18 (dark purple).
