# Supplementary material for: Copper-Arsenic-Sulfide Thin-Films from Local Raw Materials Deposited via RF Co-Sputtering for Photovoltaics
Source: Nanomaterials (Basel). 2022 Sep 20;12(19):3268. doi: 10.3390/nano12193268 (PMC9565231; doi:10.3390/nano12193268)
Supplement: Supplementary file 1 [file nanomaterials-12-03268-s001.zip › nanomaterials-1884142-supplementary.pdf]

Supplementary Material

Optoelectronic properties of copper-arsenic-sulfide thin-films deposited via RF co-sputtering for photovoltaics

Pedro Centeno[1]*, Miguel Alexandre[1], Filipe Neves[2], Elvira Fortunato[1], Rodrigo Martins[1], Hugo Águas[1]*, Manuel J. Mendes[1]*

Affiliation:

[1] i3N/CENIMAT, Department of Materials Science, Faculty of Science and Technology, NOVA University Lisbon and CEMOP/UNINOVA, Campus de Caparica, Caparica 2829-516, Portugal

[2] LNEG, Laboratório Nacional de Energia e Geologia, Estrada do Paço do Lumiar, 22, 1649-038 Lisboa, Portugal

SEM-EDS analysis

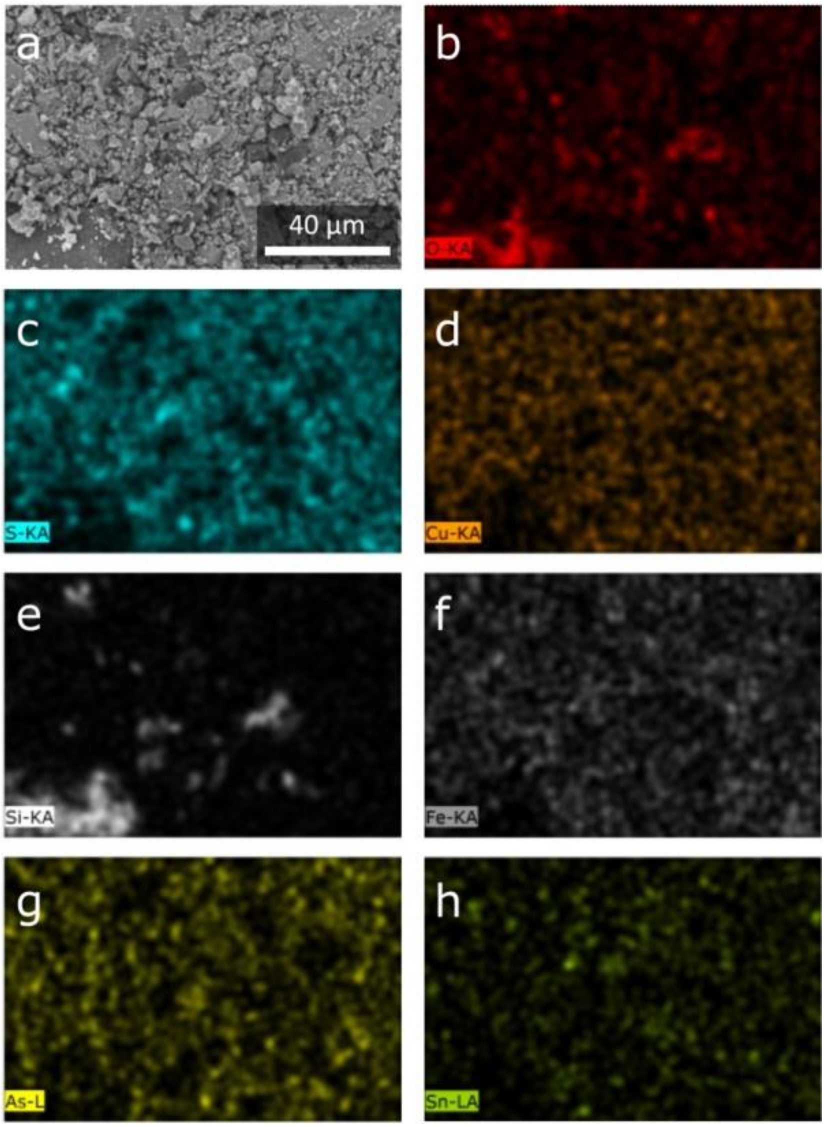

Figure S 1. (a) SEM image of copper-arsenic-sulfide target used in the deposition of the CAS films and (b)-(h) respective EDS color map images of the target displaying its content: Copper, arsenic and sulfur with SiO_2, Fe and Sn impurities. The scale bar is common to all images.

SEM-EDS calculations

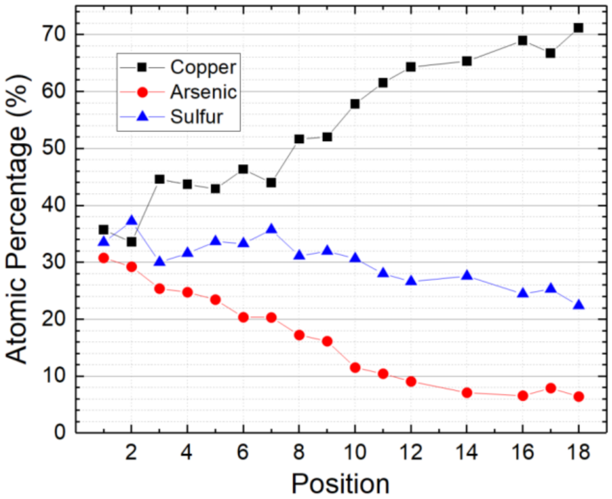

Figure S 2. Atomic percentages of Cu, As and S in each position within the thin-film.

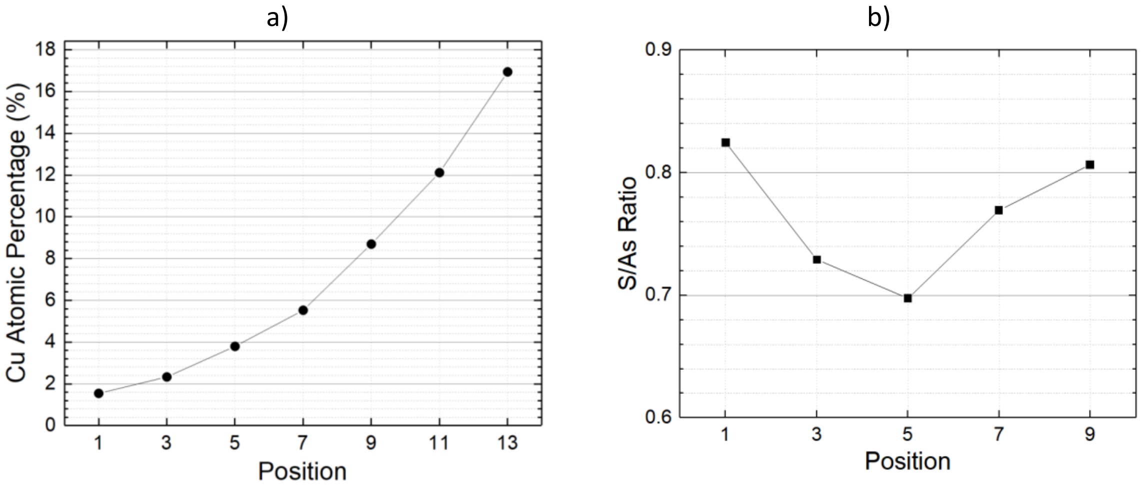

Figure S 3. (a) Cu atomic percentage obtained in a preliminary 20 by 10 cm Cu thin-film, deposited with the same parameters of the main deposition (50W, 50sccm Ar flow, 1.0 mTorr). (b) S/As Ratio of preliminary 10 by 10 cm As-S thin-film, deposited with the same parameters of the main deposition (50W, 50sccm Ar flow, 1.0 mTorr). The positions presented correspond to those illustrated in Figure 1 of the main article.

XRD analysis

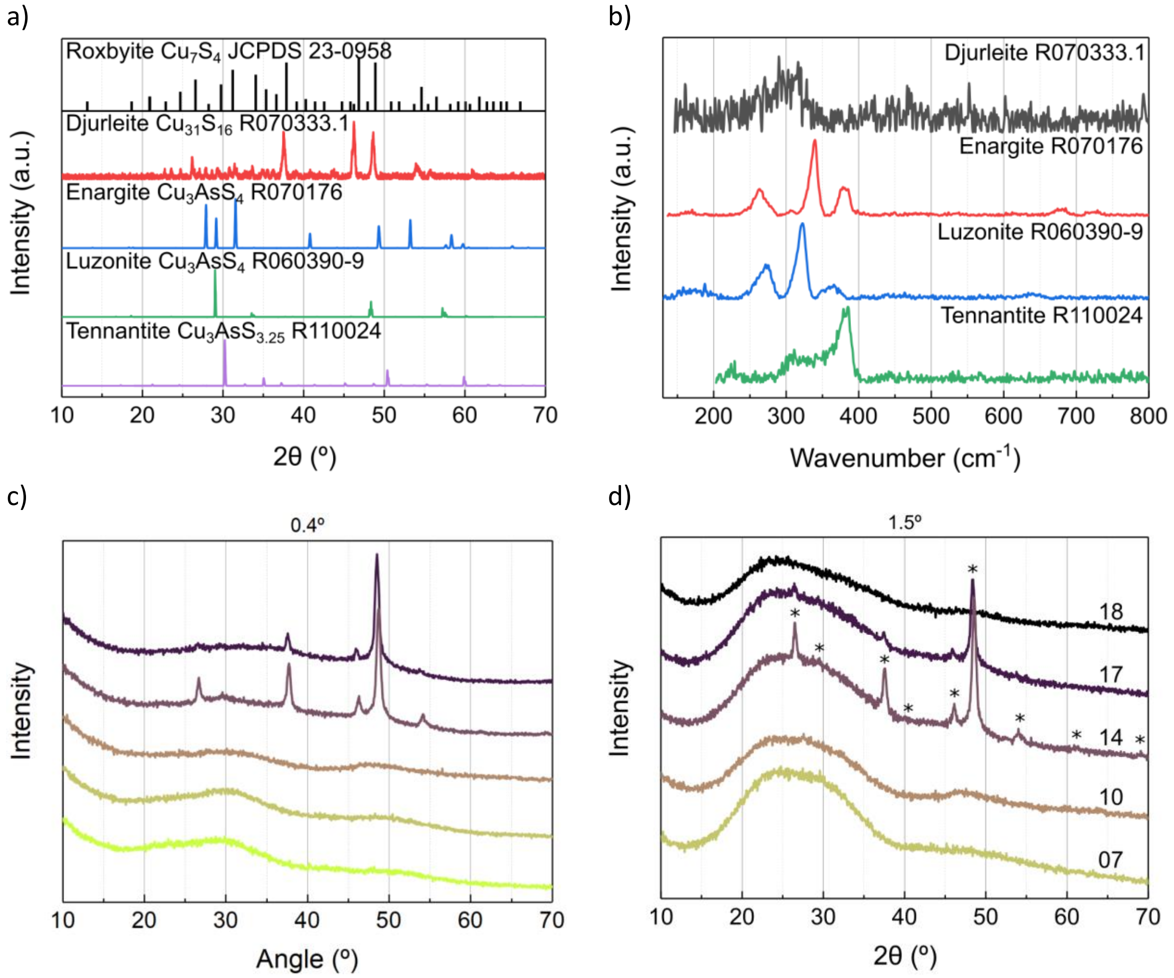

Figure S 4. (a) XRD reference spectra of two copper sulfide phases (Roxbyite and Djurleite) and three copper arsenic sulfide phases (Enargite, Luzonite and Tennantite). (b) Raman reference spectra of Djurleite and three copper arsenic sulfide phases (Enargite, Luzonite and Tennantite). GIXRD spectra of five positions within the deposited thin-film with Omega = 0.4 º (c) and 1.5 º (d).

Optical analysis

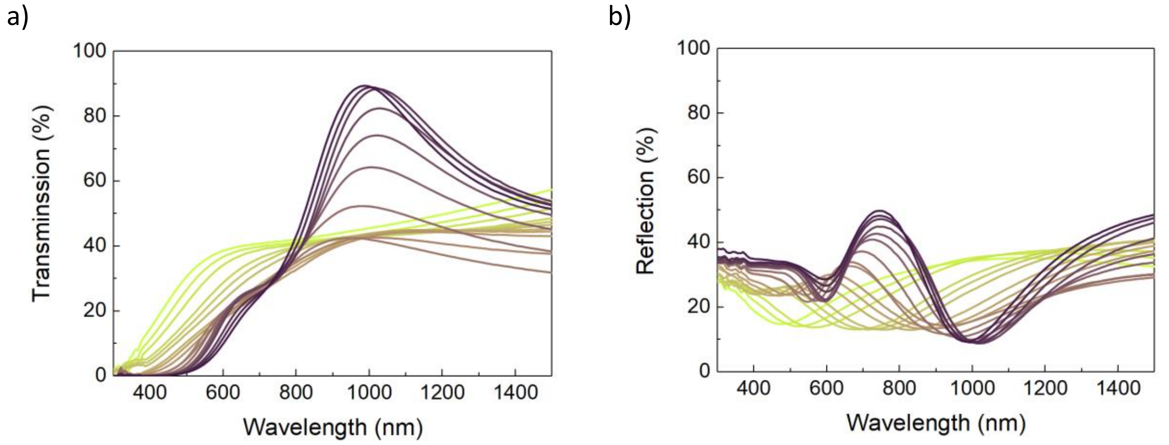

Figure S 5. Measured transmission (a) and reflection (b) spectra of positions 1 (light yellow) to 18 (dark purple).
